# Myocardial Scar Identified by Magnetic Resonance Imaging Can Predict Left Ventricular Functional Improvement after Coronary Artery Bypass Grafting

**DOI:** 10.1371/journal.pone.0081991

**Published:** 2013-12-16

**Authors:** Tao Yang, Min-Jie Lu, Han-Song Sun, Yue Tang, Shi-Wei Pan, Shi-Hua Zhao

**Affiliations:** 1 Department of Cardiovascular Surgery, State Key Laboratory of Cardiovascular Disease, Fu Wai Hospital and Cardiovascular Institute, National Center for Cardiovascular Diseases, Peking Union Medical College and Chinese Academy of Medical Sciences, Beijing, China; 2 Department of Radiology, State Key Laboratory of Cardiovascular Disease, Fu Wai Hospital and Cardiovascular Institute, National Center for Cardiovascular Diseases, Peking Union Medical College and Chinese Academy of Medical Sciences, Beijing, China; University of Louisville, United States of America

## Abstract

**Background:**

Previous studies have shown that viable myocardium predicts recovery of left ventricular (LV) dysfunction after revascularization. Our aim was to evaluate the prognostic value of myocardial scar assessed by late gadolinium-enhanced cardiovascular magnetic resonance imaging (LGE-CMR) on functional recovery in patients undergoing coronary artery bypass grafting (CABG).

**Methods:**

From November 2009 to September 2012, 63 patients with reduced left ventricular ejection fraction (LVEF) referred for first-time isolated CABG were prospectively enrolled, 52 were included in final analysis. LV functional parameters and scar tissue were assessed by LGE-CMR at baseline and 6 months after surgery. Patency of grafts was evaluated by computed tomography angiography (CTA) 6 months post-CABG. Predictors for global functional recovery were analyzed.

**Results:**

The baseline LVEF was 32.7±9.2%, which improved to 41.6±11.0% 6 months later and 32/52 patients improved LVEF by ≥5%. Multivariate logistic regression analysis showed that the most significant negative predictor for global functional recovery was the number of scar segments (Odds ratio 2.864, 95% Confidence Interval 1.172–6.996, p = 0.021). Receiver-Operator-Characteristic (ROC) analysis demonstrated that ≤4 scar segments predicted global functional recovery with a sensitivity and specificity of 85.0% and 87.5%, respectively (AUC = 0.91, p<0.001). Comparison of ROC curves also indicated that scar tissue was superior to viable myocardium in predicting cardiac functional recovery (p<0.001).

**Conclusions:**

Our findings indicated that scar tissue on LGE-CMR is an independent negative predictor of cardiac functional recovery in patients with impaired LV function undergoing CABG. These observations may be helpful for clinicians and cardiovascular surgeons to determine which patients are most likely to benefit from surgical revascularization.

## Introduction

Coronary artery bypass grafting (CABG) has been shown to improve both symptoms and prognosis in patients with coronary artery disease (CAD) and left ventricular (LV) dysfunction. Although surgical risk in patients with poor left ventricular ejection fraction (LVEF) is relatively higher, these patients could benefit greatly from CABG [1]. Previous studies have linked improvement in cardiac function or survival after CABG to the presence of dysfunctional but viable myocardium [2], [3], [4], [5]. In 2011, however, the Surgical Treatment for Ischemic Heart Failure (STICH) trial [6] demonstrated that the assessment of myocardial viability did not identify patients with a differential survival benefit from surgical revascularization, as compared with medical therapy alone. Other studies also indicated that to include only viable myocardium in a studying algorithm has limitations in predicting global functional improvement and not all patients with ischemic cardiomyopathy recover in function after successful revascularization, despite the presence of viable myocardium identified using various imaging protocols [7], [8].

Late gadolinium-enhanced cardiovascular magnetic resonance imaging (LGE-CMR) has emerged as an accurate and reproducible modality for detection and quantification of irreversibly damaged myocardial scar over the past few years [9]. Furthermore, CMR could define the transmural extent of scar tissue (LGE-CMR), and also make comprehensive assessment of LV function as well as volume (cine-CMR), each of which is relevant to evaluating patients prognosis [10], [11]. Thus, the versatility of CMR makes it an increasingly powerful tool for the complete assessment of patients with ischemic cardiomyopathy.

Although LGE-CMR protocols often assess and report all of these parameters, previous studies have evaluated the value of scar tissue to predict patients' outcomes and demonstrated that presence of LGE could identify patients with higher risk of events [12], [13], [14]. We also found that prior studies focused the analysis portion more on the viable myocardium [2], [3], [15], [16], yet not directly correlated scar tissue with late functional improvement after surgical revascularization. Therefore, it is unknown to what extent myocardial scar affects cardiac functional recovery. Taking advantage of this cohort of patients, our aim was to assess the prognostic power of myocardial scar and volumes over patient clinical data for the prediction of cardiac functional recovery.

## Materials and Methods

### Study Population

**Figure 1:**
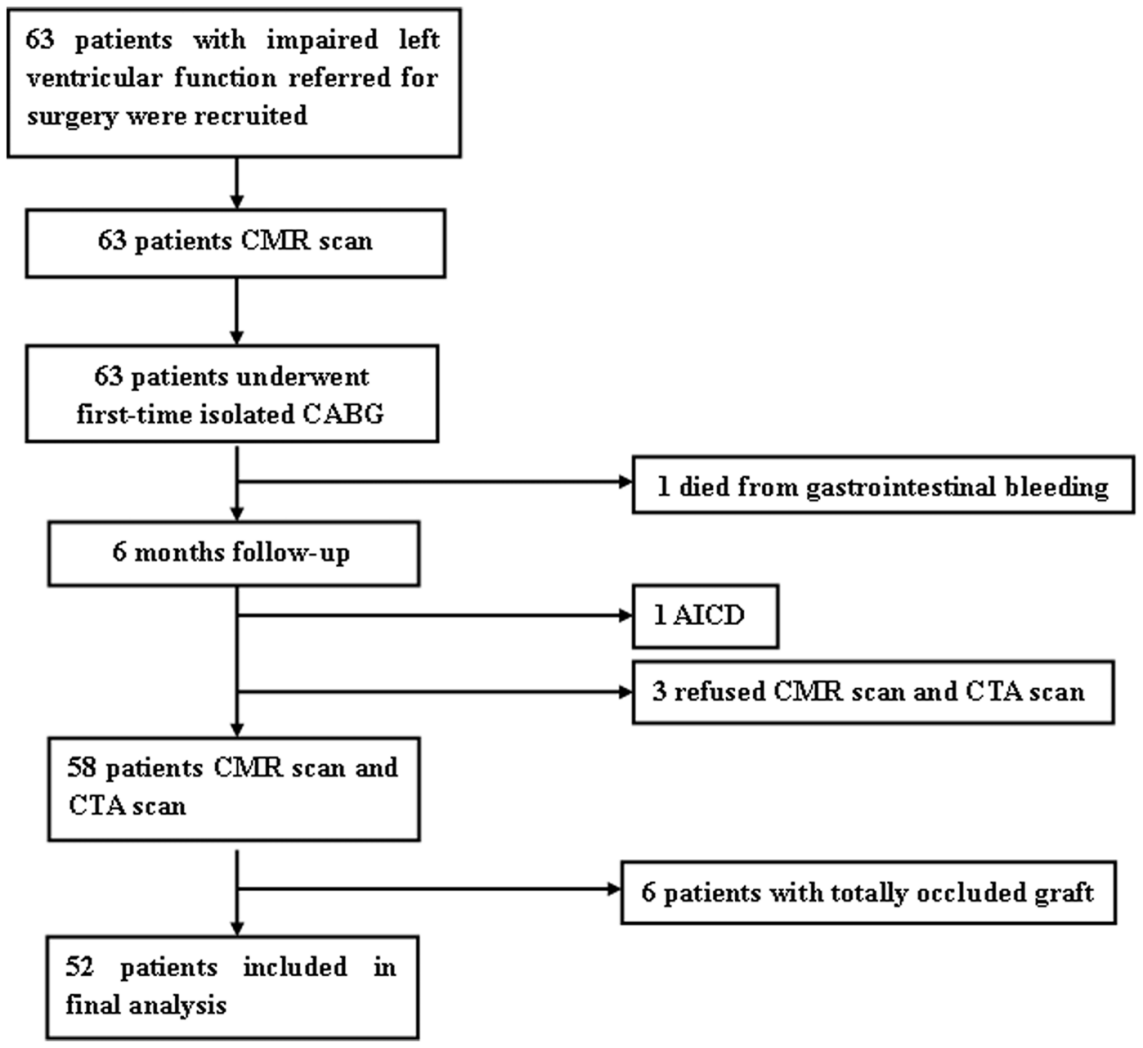
Flow Diagram of Patients Recruitment. AICD, automated implantable cardiac defibrillator; CABG, coronary artery bypass grafting; CMR, cardiovascular magnetic resonance imaging; CTA, computed tomography angiography.

Consecutive patients with CAD and LV dysfunction referred to Department of Cardiovascular Surgery at our hospital for first-time isolated CABG between November 2009 and September 2012 were prospectively enrolled. Patients were selected if they had previous Q-wave myocardial infarction (MI) on electrocardiogram(ECG); had abnormalities in regional wall motion on either contrast ventriculography or resting echocardiography; New York Heart Association (NYHA) function class of ≥II, LVEF<50% by CMR. Patients were excluded if they had valve disease (significant mitral or aortic insufficiency or stenosis), a history of MI<3 months before surgery; unstable angina and CMR contraindications (eg, noncompatible biometallic implants, claustrophobia or malignant arrhythmias). As a result, we identified 63 patients eligible who underwent an imaging protocol combining three components: left ventricular volumes and function, myocardial LGE and segmental wall motion. LGE-CMR was performed before and 6 months after CABG, computed tomography angiography (CTA) scan was also performed 6 months later to evaluate the grafts patency. Finally, 11 patients were excluded, including 6 with graft failure and 5 for other reasons, leaving a study population of 52 patients (Figure 1). This study was approved by the institutional review board of Fu Wai hospital and all patients gave written informed consent.

### CMR Protocol

All CMR examinations were performed using a 1.5 Tesla CMR scanner (Avanto, Siemens AG, Germany), using wireless vector ECG gating. LV end-diastolic volumes (LVEDV) and LV end-systolic volumes (LVESV) were determined for calculation of LVEF with breath-hold steady-state free precession (SSFP) cine sequence. The main sequence parameters were as follows: TR = R-R interval, TE 1.3 ms, flip angle 60° to 70°, matrix 192×156, slice thickness 6 mm, field of view 300–380 mm. The end-diastolic, end-inspiration 4-chamber and 2-chamber views served as the reference images to plan the short-axis slices. A phase-sensitive inversion-recovery gradient-echo pulse sequence with coil intensity correction was used for LGE imaging 10–15 minutes after a 0.2 mmol/kg intravenous dose of Gd-DTPA (gadopentate dimeglumine, Magnevistw, Bayer Healthcare Pharmaceuticals, Wayne, NJ, USA) during breath hold in a series of short-axis planes and a 4- and 2-chamber long-axis plane. Parameters for LGE imaging were as follows: TR 700 ms, TE 3.4 ms, flip angle 25°.

### Imaging Analysis

Cardiac function was analyzed with Argus software (Siemens AG, Munich, Germany) by two experienced radiologists blinded to clinical data. Determination of end-diastole and end-systole was visually achieved and allowed calculation of LV mass, LVEDV, LVESV, from which LVEF was derived. The LV mass was calculated by subtracting endocardial from epicardial volume at end-diastole and multiplying by 1.05 g/cm^3^.

LGE images were evaluated by the same experienced radiologists using an identical 17-segment model [17]. Hyperenhanced areas resembling LGE were determined as areas with signal intensities >2SDs above remote normal myocardial region. A five-point scale system was used to describe the transmural extent of LGE in each of the segment (scar score): 0 = no LGE, 1 = 1%–25% LGE, 2 = 26%–50% LGE, 3 = 51%–75% LGE, and 4 = 76%–100% LGE. If there was no agreement in the interpretations, the image was reevaluated by two radiologists until a consensus was reached. A segment was considered “viable” when the transmural extent of LGE was between 1% and 50%, “scar” when the transmural extent of LGE was between 51% and 100%. The cut-off value of 50% LGE was the best threshold to define segmental viability for the purpose of predicting recovery of cardiac function [2], [7], [13]. Additionally, to quantify the extent of scar tissue, the following definitions were used [13]: 1) spatial extent, the number of affected segments; 2) normal segments, the number of segments with a scar score of 0 (or no LGE); 3) viable segments, the number of segments with a scar score of 1 or 2; 4) scar segments, the number of segments with a scar score of 3 or 4; 5) total scar score, summed segmental scar scores per patient. The severity of segmental wall motion was determined on a four-point scale system: 0 = normal, 1 = hypokinesis, 2 = akinesis, 3 = dyskinesis. Wall motion score (WMS) was calculated as the sum of the individual scores of 17 segments in each patient. The change in WMS (ΔWMS) and LVEF (ΔLVEF) was also recorded.

In patients who had successful revascularization, an improvement in segmental wall motion by ≥1 grade was considered significant and ΔLVEF≥5% was used to define global functional recovery (responders), a reductions of 10% or more in LVEDV and LVESV were also considered clinically meaningful reverse remodeling [18], [19].

### Treatment and Procedures

All surgeries were performed by the same surgical team experienced in both on-pump and off-pump CABG. The aim of surgery was to obtain complete revascularization. The left internal mammary artery and great saphenous vein were harvested in each patient and quality of grafts was assessed intra-operatively with the use of a transit-time flow probe (Medi-stim Butterfly flowmeter, Oslo, Norway).

### Follow-up

The final 52 patients were followed up for cardiovascular events (CVEs) occurred from the first day after discharge to 38 months of the follow-up: ventricular arrhythmia (tachycardia or fibrillation), heart failure, recurrence of angina, MI, repeat revascularization, and cardiac death, with telephone contact with the patients, their relatives, or from review of medical records. No patients were lost to follow-up.

### Statistical Analysis

Statistical analysis was performed by using SPSS v13.0 (SPSS Inc., Chicago, Illinois). Continuous variables were presented as mean ± standard deviation (SD) or median (interquartile range, 25% to 75%). Categorical variables were reported as absolute numbers and percentages. Between-group comparisons were performed with χ^2^ test or Fisher's exact test for categorical variables and Student's *t* test for continuous variables that were distributed normally. Non-normally distributed continuous variables were compared by the Mann-Whitney U test. All tests were 2-sided. Any p value of less than 0.05 was considered statistically significant.

For prediction of global functional recovery, univariate and multivariate logistic regression analysis was performed to determine which variables were the best predictors. Variables entered the multivariate stage that had p<0.1 in the univariate analysis and then were selected in ‘enter’ method at a significance level of 0.05. Assumptions checked for multivariate logistic regression were: no interaction, linearity (for continuous explanatory variables only) and independency of observations. Receiver-operating-characteristic (ROC) analysis was performed to assess the most appropriate cut-off value of the predictor derived from the logistic regression analysis and the percent of segments with wall motion improvement which best indicates global functional recovery.

After adjustment for multiple confounders, only the number of scar segments as determined by LGE-CMR appeared significantly and negatively related with functional recovery. ROC analysis also indicated that the presence of four or less scar segments could predict significant recovery of global function with the optimal sensitivity and specificity. Therefore, patients were stratified into two groups (≤4scar segments n = 31, >4 scar segments n = 21) according to the extent of scar tissue on LGE-CMR. The effect of surgery was compared using a paired t-test. Kaplan-Meier survival analysis was used to further analyze CVE-free survival of both cohorts, and comparisons between survival distributions were made using log-rank test.

## Results

### Study Population and Outcomes of CABG

Patients enrolled and excluded from our study are presented in Figure 1. Data analysis was based on the final 52 patients whose grafts were all patent evidenced by CTA scanning (Patients with graft failure were excluded from our study, Figure 1). There were 44 men and 8 women with an average age of 58.0±8.4 years. The baseline LVEF was 32.7%±9.2% and 30 (57.7%) of these subjects had LVEF≤35%. Patients were stratified into two groups: responders (patients with ΔLVEF≥5%) and non-responders (patients with ΔLVEF<5%). Baseline characteristics are shown in Table 1. There was no significant difference in clinical parameters between these two groups. The periprocedural data and CABG outcomes are given in Table 2. No significant differences in the number of bypass grafts, operation time, ventilation time, length of hospital stay, or type of surgery were observed and the incidences of postoperative complications were also similar.

**Table 1:** Baseline characteristics of the patients.

Variables	All patients	Responders*	Non-responders	P value†
	(n = 52)	(n = 32)	(n = 20)	
**Age, years**	58.0±8.4	59.6±8.5	56.4±6.8	0.163
**Female**	8(15.4)	5(15.6)	3(15.0)	1.000
**Hypertension**	26(50.0)	17(53.1)	9(45.0)	0.569
**Diabetes mellitus**	21(40.4)	15(46.9)	6(30.0)	0.228
**Hypercholesterolemia**	36(69.2)	23(71.9)	13(65.0)	0.601
**Brain infarction history**	5(9.6)	2(6.3)	3(15.0)	0.577
**Current smoker**	39(75.0)	24(75.0)	15(75.0)	1.000
**Family history of CAD**	27(51.9)	16(50.0)	11(55.0)	0.726
**Angiographic findings**				
**(stenosis>50%)**				
**Two-vessel disease**	12(23.1)	8(25.0)	4(20.0)	0.938
**Three-vessel disease**	40(76.9)	24(75.0)	16(80.0)	
**LVEF≤35%**	30(57.7)	21(65.6)	9(45.0)	0.143
**NYHA class III/IV**	18(34.6)	10(31.3)	8(40.0)	0.986
**Medication on discharge**				
**Aspirin**	52(100)	32(100)	20(100)	1.000
**ACE inhibitor**	35(67.3)	21(65.6)	14(70.0)	0.744
**β-blocker**	49(94.2)	30(93.8)	19(95.0)	1.000
**Statin**	52(100)	32(100)	20(100)	1.000
**CCB**	26(50.0)	15(46.9)	11(55.0)	0.569

± SD or n (%). Values are expressed as mean

≥5%. Responders were defined as improvement in LVEF

†P value for Responders and Non-responders.

ACE, angiotensin-converting enzyme; CAD, coronary artery disease; CCB, calcium channel blockers; LVEF, left ventricular ejection fraction; NYHA, New York Heart Association.

**Table 2:** Periprocedural data and CABG outcomes.

Variables	All patients	Responders	Non-responders	P value
	(n = 52)	(n = 32)	(n = 20)	
**Operation time,min**	236.8±68.1	246.3±70.3	221.8±63.2	0.210
**Ventilation time, h**	17.0(14.3,21.8)	19.5(15.0,21.0)	16.0(13.0,23.8)	0.341
**ICU stay, h**	69.5(38.5,92.8)	70.0(28.0,93.5)	68.5(39.5,92.3)	0.792
**Hospital stay,days**	8.0 (7.0, 10.0)	9.0 (8.0, 10.0)	8.0 (7.0, 12.0)	0.539
**Grafts per patient**	3.3±0.7	3.4±0.7	3.3±0.7	0.523
**Off-pump surgery**	24(46.2)	15(46.9)	9(45.0)	0.895
**Outcomes**				
**New-onset AF**	3(5.8)	2(6.3)	1(5.0)	1.000
**VA**	2(3.8)	0(0)	2(10.0)	0.279
**LCOS**	1(1.9)	0(0)	1(5.0)	0.385

Values are expressed as mean ± SD, median (interquartile range) or n(%).

AF, atrial fibrillation; ICU, intensive care unit; LCOS, low cardiac output syndrome; VA, ventricular arrhythmia.

### CMR Findings

The CMR results are summarized in Table 3. No significant difference was found between responders and non-responders in global LV function or volume at baseline. For myocardial viability, three parameters with significant differences were the number of viable segments, the number of scar segments and total scar score. Moreover, ΔWMS was significantly lower in non-responders, but no significant difference in WMS at baseline was found. Patients with ΔLVEF≥10% and percent of segments with wall motion improvement were both different between the two groups (p<0.001).

**Table 3:** Cardiovascular Magnetic Resonance Imaging Data.

Baseline LV parameters	All patients	Responders	Non-responders	P value
	(n = 52)	(n = 32)	(n = 20)	
**LVEF,%**	32.7±9.2	32.1±9.0	33.7±9.8	0.548
**LVEDD, mm**	59.8±7.0	60.1±7.3	59.2.±6.8	0.641
**LVEDVI, ml·m^−2^**	100.8±30.1	102.6±30.5	97.9±29.9	0.589
**LVESVI, ml·m^−2^**	68.6±28.4	70.2±28.1	66.1±29.5	0.617
**CI, L · min^−1^ · m^−2^**	2.2±0.6	2.3±0.6	2.2±0.4	0.727
**Dysfunctional segments**	12.8±3.6	12.7±3.8	12.8±3.2	0.937
**WMS**	18.3±6.1	18.3±6.0	18.3±6.5	0.992
**Myocardial viability**				
**Spatial extent**	10.5±3.0	9.9±3.3	11.5±2.1	0.065
**Viable segments**	6.8±3.0	7.5±3.3	5.7±2.0	0.035
**Scar segments**	3.6±2.0	2.6±1.5	5.4±1.3	<0.001
**Total Scar Score**	23.0±7.8	19.8±7.4	28.1±5.6	<0.001
**Functional Improvement**				
**ΔWMS** *	−5.0 (−8.0,−3.0)	−7.0(−9.0,−4.2)	−3.0(−5.5,−1.0)	0.001
**ΔLVEF** †	8.4 (3.2, 13.7)	11.7(9.4,16.0)	2.1 (0, 4.2)	<0.001
**ΔLVEF≥10%**	20(38.5)	20(62.5)	0(0)	<0.001
**Percent of segments with wall motion improvement (%)** ‡	42.0±18.8	52.9±14.0	24.6±10.2	<0.001

Values are expressed as mean ± SD, median (interquartile range) or n (%).

* ΔWMS, change in WMS.

†ΔLVEF, change in LVEF.

‡ Percent of segments with wall motion improvement: number of segments with improved contractility divided by total analyzable segments.

CI, cardiac index; LV, left ventricular; LVEDD, left ventricular end-diastolic diameter; LVEDVI, left ventricular end-diastolic volume index; LVEF, left ventricular ejection fraction; LVESVI, left ventricular end-systolic volume index; WMS, wall motion score.

### Prediction of segmental functional recovery

**Figure 2:**
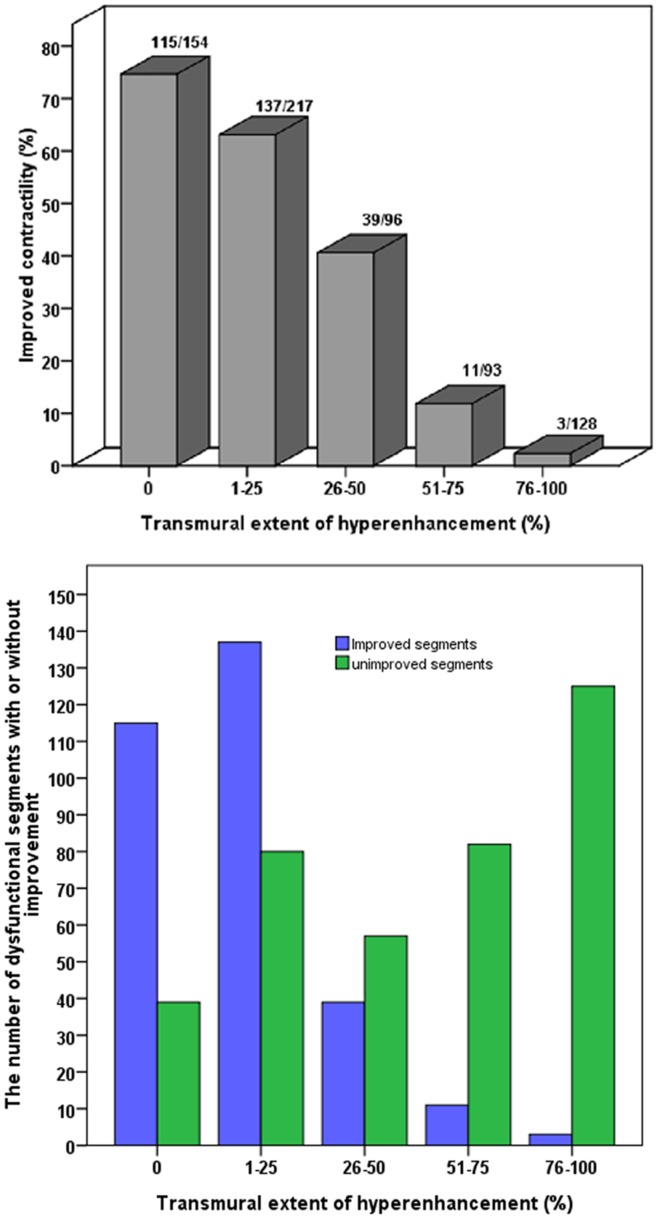
Relationship Between Transmural Extent of Scar Tissue and Functional Recovery. The extent of LGE correlates inversely with improved myocardial contractility following revascularization (top). The number of dysfunctional segments with or without improvement in different transmural extent of LGE (bottom). LGE, late gadolinium enhancement.

A total of 884 segments were analyzed, of which 688 (77.8%) segments were determined to be dysfunctional preoperatively, including 534(60.4%) segments with various degree of LGE. Six months after CABG, 305 (44.3%) segments showed functional improvement. In viable and normal segments (≤50% LGE), 291/467(62.3%) improved contraction, while in scar segments (>50% LGE), only 14/221(6.3%) improved contraction post-surgery (p<0.001). In dysfunctional segments, the extent of LGE correlates inversely with improved myocardial contractility following revascularization (p<0.001, Figure 2). This relationship was present irrespective of the severity of preoperative segmental dysfunction.

**Figure 3:**
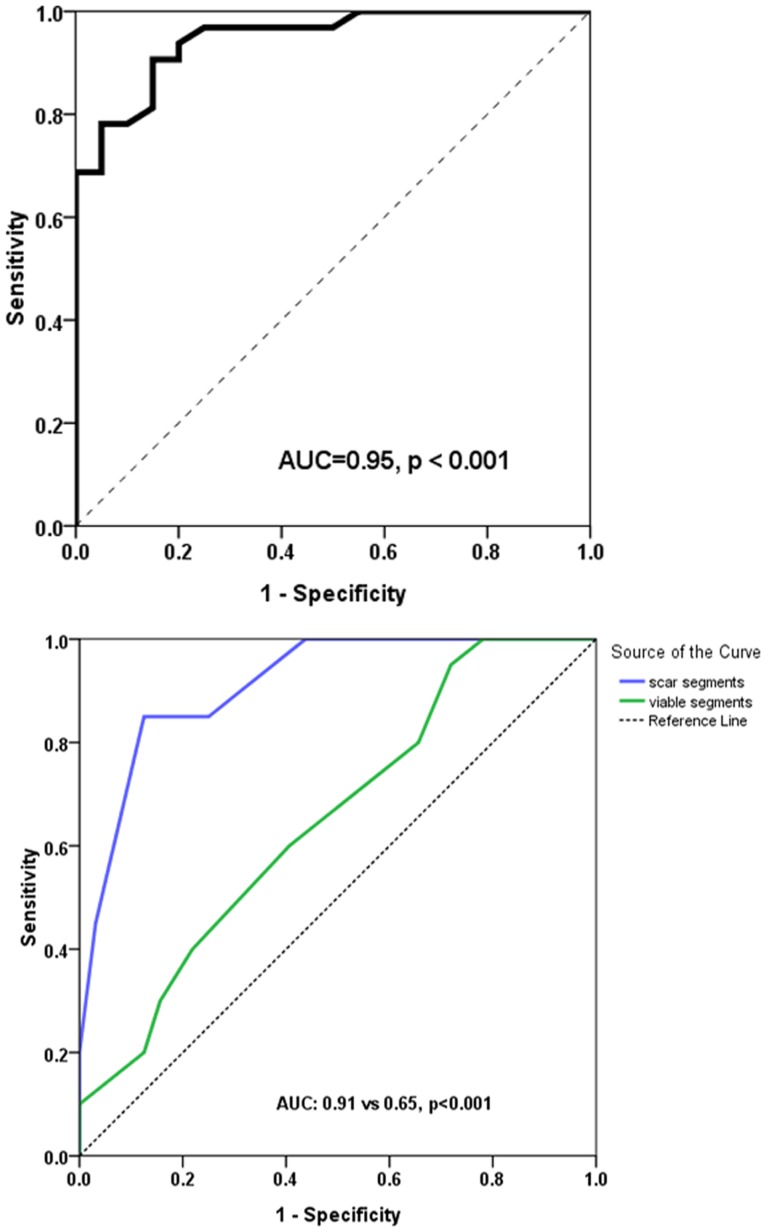
ROC Analysis of Dysfunctional Segments and Comparison of ROC Curves Between Scar and Viable Segments. ROC analysis demonstrated ≥35.5% of dysfunctional segments with wall motion improvement predicts global functional recovery (top). Comparison of ROC curves showed that the number of scar segments (AUC = 0.91) predict global functional improvement superior to the number of viable segments (AUC = 0.65) (p<0.001) (bottom). AUC, area under the curve of the receiver-operating-characteristic. ROC, receiver-operator-characteristic.

Our results further demonstrated that responders have a higher proportion of improved segments than non-responders (52.9±14.0%vs24.6±10.2%, p<0.001) and ≥35.5% of dysfunctional segments with wall motion improvement clearly predicts global functional recovery, with a sensitivity of 90.6% and specificity of 85.0% (AUC = 0.95, p<0.001) (Figure 3).

### Prediction of global functional recovery

**Figure 4:**
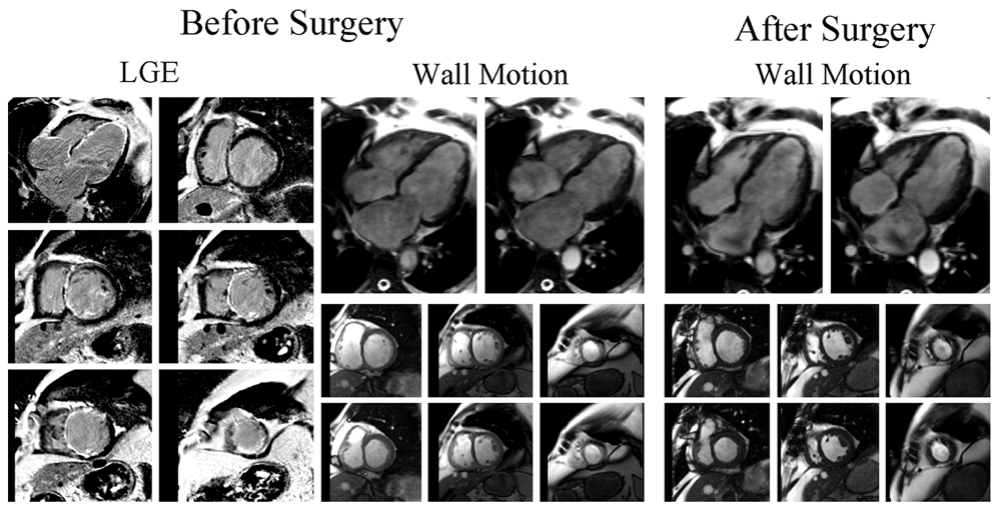
Patient without Global Functional Recovery. LGE and wall motion images before and after CABG of a 56-year-old man with 7 scar segments who showed no evidence of global functional recovery and reverse remodeling post-surgery. CABG, coronary artery bypass grafting. LGE, late gadolinium enhancement.

**Figure 5:**
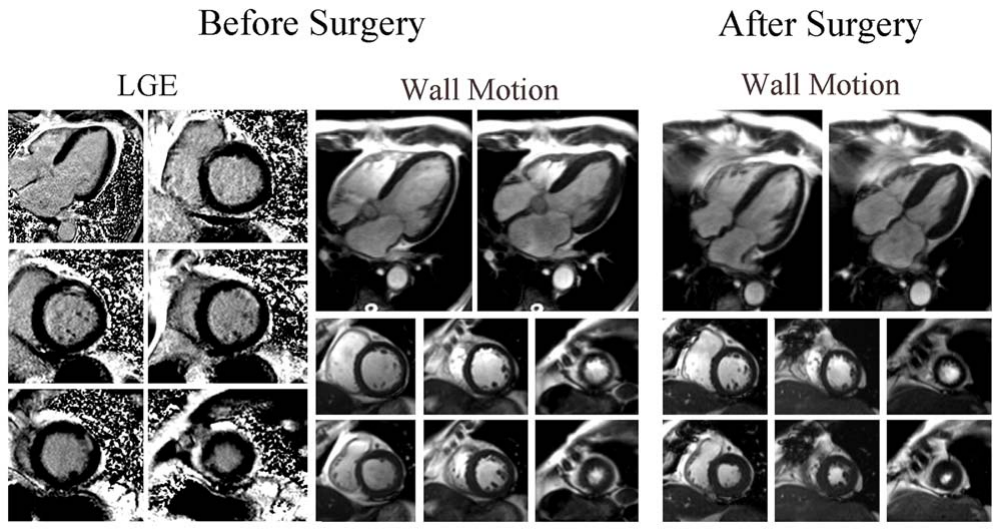
Patient with Global Functional Recovery. LGE and wall motion images before and after CABG of a 69-year-old man with 2 scar segments who had improvement in both global function and LV geometry.

Firstly, univariate analysis demonstrated that the number of viable segments, scar segments and total scar score are predictors of global functional recovery. However, by using multivariate logistic analysis, only the number of scar segments was shown to be negatively associated with global functional recovery post-CABG (Odds Ratio 2.864, 95% Confidence Interval 1.172–6.996, p = 0.021 Table 4). Patients with substantial scar tissue on LGE-CMR showed no evidence of functional improvement (Figure 4 and Movie S1), whereas global functional recovery was demonstrated in patients with small scar area (Figure 5 and Movie S2).

**Table 4:** Univariate and multivariate analyses for the prediction of global functional recovery following surgical revascularization.

Univariate	Odds Ratio	95% Confidence Interval	P value
**Spatial extent**	1.211	0.984–1.492	0.071
**Viable segments**	0.774	0.603–0.995	0.045
**Scar segments**	3.506	1.856–6.622	<0.001
**Total Scar score**	1.214	1.081–1.364	0.001

Multivariate	Odds Ratio	95% Confidence Interval	P value
**Scar segments**	2.864	1.172–6.996	0.021

ROC analysis indicated ≤4scar segments demonstrated the optimal sensitivity of 85.0% and specificity of 87.5% for predicting global functional recovery (AUC = 0.91, p<0.001). In addition, the positive and negative predictive values were 87.9% and 84.5%, respectively. Comparison of ROC curves also showed that the number of scar segments can predict global functional recovery superior to the number of viable segments (AUC 0.91 vs 0.65, p<0.001, Figure 3).

**Figure 6:**
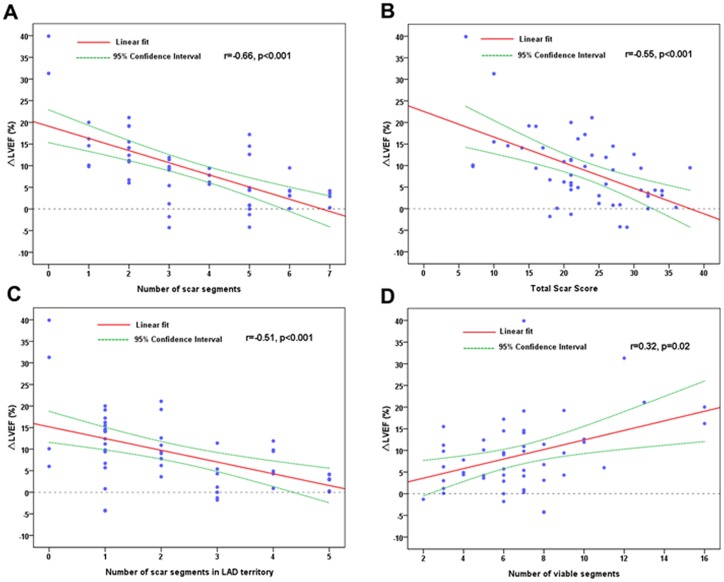
The Association Between Myocardial Viability and Change in LVEF. Scatter plot showing the linear association between the number of scar segments(A), total scar score(B), the number of scar segments in LAD territory(C), the number of viable segments(D) and change in LVEF (ΔLVEF) at 6 months post-surgery. LAD, left anterior descending coronary artery. LVEF, left ventricular ejection fraction.

There was a significant linear association between preoperative LVEF and WMS for each individual (r = −0.75, p<0.001). Furthermore, we also found that the number of scar segments, total scar score and the number of scar segments in left anterior descending coronary artery (LAD) territory correlated very well with ΔLVEF after CABG (Figure 6). A lesser association was presented when the number of viable segments was considered (Figure 6).

### Follow-up

After multivariate logistic analysis, patients were divided into two groups (≤4 scar segments, n = 31, Group A and >4 scar segments, n = 21, Group B) according to the number of scar segments. Patients with ≤4 scar segments showed significant improvement in both global function and reverse remodeling. There was also statistical improvement in both LVEF and LV geometry in patients with >4 scar segments. However, the mean difference of the improvement was less remarkable than that of patients with ≤4 scar segments [4.2 (1.9 to 6.6)% vs12.0 (8.7 to 15.2)%, p = 0.001] (Table 5).

**Table 5:** Functional Improvement in patients subsequent to CABG (n = 52).

	Baseline	6 months	Mean Difference, 95%CI	P value*
**≤4 scar segments**				
**(n = 31)**				
**LVEF (%)**	34.1±8.5	46.1±10.0	12.0 (8.7 to 15.2)	<0.001
**LVEDD (mm)**	60.0±7.1	52.9±6.0	−7.1 (−9.2 to −5.0)	<0.001
**LVEDVI (ml·m^−2^)**	98.4±26.5	74.7±23.9	−23.8 (−31.3 to −16.2)	<0.001
**LVESVI (ml·m^−2^)**	64.7±24.3	41.7±21.3	−23.0 (−29.6 to −16.4)	<0.001
**LVMI (g·m^−2^)**	58.9±18.8	49.5±14.2	−9.4 (−14.8 to −4.0)	0.001
**>4 scar segments**				
**(n = 21)**				
**LVEF (%)**	30.7±10.1	35.0±8.9	4.2 (1.9 to 6.6)	0.001
**LVEDD (mm)**	59.3±7.2	55.9±7.6	−3.4 (−5.6 to −1.1)	0.005
**LVEDVI (ml·m^−2^)**	104.3±35.1	94.3±24.4	−10.0 (−18.9 to −1.0)	0.031
**LVESVI (ml·m^−2^)**	74.4±33.3	62.1±23.5	−12.4 (−20.6 to −4.1)	0.005
**LVMI (g·m^−2^)**	54.9±18.2	49.8±14.3	−5.1 (−11.7 to −1.4)	0.116

Values are expressed as mean ± SD or mean (95% CI).

P Value* for baseline and 6 months.

CI, confidence interval; LVMI, left ventricular mass index.

Other abbreviations as in Table 3.

**Figure 7:**
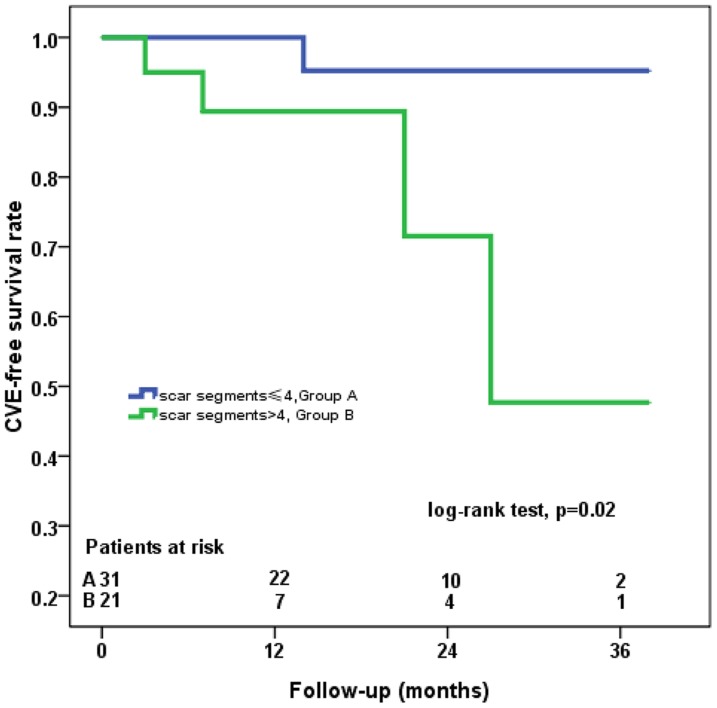
Prognostic Value of Myocardial Scar. Kaplan-Meier curve showing that the CVE-free survival rate was significantly worse in patients with>4 scar segments on LGE-CMR. CVE, cardiovascular events. LGE-CMR, late gadolinium-enhanced cardiovascular magnetic resonance imaging.

The median follow-up time was 14.0 months (range, 6–38 months) and no death occurred. Patients with ≤4 scar segments had a higher proportion of NYHA I class compared to patients with >4 scar segments (93.5% vs 66.7%, p = 0.03). During follow-up time, one patient required rehospitalization due to heart failure (Group B), one patient had recurrence of angina (Group B), one patient had nonfatal MI (Group A) and two patients suffered from ventricular arrhythmia (Group B). Furthermore, the mid-term CVE-free survival rate was significantly higher in patients with ≤4 scar segments (log-rank test, p = 0.02, Figure 7).

## Discussion

The salient findings of this study were that the number of scar segments was the best independent negative predictor of global functional recovery with perfect sensitivity and specificity and had a significant linear association with improvement in LVEF, suggesting that substantial scar tissue often revealed a poor response to CABG. These observations may be useful for pre-operative selection of patients for revascularization in everyday practice.

In the present study, not only quality of grafts was assessed intra-operatively with the use of a transit-time flow probe, follow-up CTA scan was also undertaken to evaluate grafts patency as part of this study. Furthermore, patients with totally occluded grafts were excluded. Thus, grafts patency can be definitely excluded as a confounding factor in predicting cardiac functional improvement, which makes the current study more convincing.

### LGE-CMR for prediction of segmental functional recovery

The current study showed that the transmural extent of myocardial scar is inversely associated with functional improvement of myocardium and it is highly predictive for segmental functional recovery after surgical revascularization, which was consistent with previous reports [2], [7], [15].In our study, we found that only 62% of dysfunctional segments without evidence of scar improved contractility. However, Kim et al [2] and Selvanayagam et al [15] reported 78% and 82% of dysfunctional unscarred segments recovered in function at follow-up, respectively. Furthermore, Pegg et al [7] demonstrated 73% of segments without evidence of scar had functional recovery. The reason might be that our patients' mean preoperative LVEF was lower (33% vs 43% [2], 61% [15], 38% [7]), representing the worst average LV function of published studies, and with a higher prevalence of dysfunctional myocardium and scar tissue, which may explain the lower rates of segmental functional recovery.

Although Krittayaphong et al [20] demonstrated that increased contractility of at least 25% of dysfunctional segments is needed in order to improve overall LV function, our results determined that ≥35.5% of dysfunctional segments with wall motion improvement could predict global functional recovery. The difference may be related to a more severe LV dysfunction in our study. We also observed that some segments failed to demonstrate an improvement in function, despite with minimal or no LGE. The occurrence of perioperative MI might be accounted for the failing of segmental functional recovery. Moreover, tethering of viable regions to adjacent scar segments may result in viable myocardium having an incomplete response, thus no improvement presented.

### Prediction of global functional recovery by LGE-CMR

Previous studies have based prediction of global functional recovery on viable myocardium: Bax et al [21] demonstrated global functional recovery in patients with ≥4 viable segments on low-dose dobutamine echocardiography (DE), with a sensitivity and specificity of 84% and 81%, respectively. Slart et al [16] showed that ≥3 viable segments predicted overall functional recovery with a sensitivity of 87% and specificity of 85%, defined by positron emission tomography (PET). However, information on the predictive value of scar tissue for late functional improvement in patients with depressed LVEF is insufficient and comparison of ROC curves in the current study demonstrated that myocardial scar has higher accuracy in predicting improvement of function than does viable myocardium, thus introduce the concept that scar tissue may be a better predictor. We have two points to clarify this issue: First, including only viable segments in a reporting protocol for global functional recovery presumes that normally functional myocardium makes no contribution to functional recovery after CABG. Second, myocardial scar could affect the motion of surrounding myocardium, which means that myocardial scar might have a greater impact on functional recovery and also supports the notion that tethering by scar tissue may counteract the improved contraction contributed by viable myocardium, thus prevent segmental as well as global functional recovery. As a result, our data suggests that analysis should based on scar segments instead of viable segments alone, or incorporate both viable and normal segments, as Pegg et al suggested [7], because all segments are able to contribute to the end-point.

In the substudy of the STICH trial, Panza et al demonstrated that in patients with CAD and LV dysfunction, inducible myocardial ischemia did not identify those with a worse prognosis or those with greater benefit from CABG over optimal medical therapy [22]. Bonow et al also reported the finding that the presence of viable myocardium did not identify patients with a differential survival benefit after CABG, as compared with medical therapy alone [6]. The conclusion seemed counterintuitive, because the viability assessment was only based on single-photon-emission computed tomography (SPECT) and dobutamine echocardiography (DE), which pose significant limitations in their ability to detect myocardial viability [23]. From our point of view, a randomized study of surgical revascularization versus medical therapy is needed after viability evaluation with a standard imaging protocol such as LGE-CMR [2], [23], which is an accurate and powerful modality for detection and quantification of myocardial scar. Moreover, the STICH investigators focused more on viable myocardium and patients long-term survival benefit instead of scar tissue as well as recovery of cardiac function, which is, however, the focus of the current study. Bonow et al also indicated that limited data are available regarding survival benefit in patients with LV dysfunction who were studied on CMR [6]. Therefore, if the STICH trial incorporated LGE-CMR technique and defined improvement in global function after CABG as their primary endpoint, the conclusion might be quite different.

In this study, myocardial scar mass was not used for functional analysis because we considered that scar mass itself has several limitations in predicting cardiac functional recovery. Firstly and most importantly, scar burden (in grams) only takes mass of fibrosis into account, but without considering its extent and distribution, while the latter (the extent and distribution of scar tissue) may be of greater clinical significance for predicting functional recovery. For example, patient A has a larger area of subendocardial MI, while patient B has area-limited transmural MI. The total scar mass could be the same for both patients, but patient A has relatively less scar segments according to the definition of scar segments in the current study. Intuitively, prognosis of the two patients will be totally different post-CABG and patient A might have a better cardiac functional recovery. From our point of view, the semi-quantitative method (the number of scar segments) we used takes both extent and distribution of scar tissue into consideration. Secondly, the major aim of the current study is to help cardiovascular surgeons identify which patients with depressed LVEF are most likely to benefit from surgical revascularization, the segmentation of the CMR-data might be easier for them to comprehend. Thirdly, in routine practice of CMR protocol, “slice gap” during CMR scanning would somewhat affect the accurate measurement and calculation of the scar tissue (in grams), which may then affect the prediction of functional improvement and lead to lack of predictive accuracy. Our fourth consideration is that whilst there maybe merit in the mass of scar, the regional localization of this does not give a full flavour of the global cardiac involvement from infarction.

In 2004, Rizello et al [24] showed the linear association between the number of viable segments and ΔLVEF after revascularization. However, they did not tell whether there is a threshold for the number of segments that predicted global functional recovery. In the current study, we found that ΔLVEF is linearly and negatively related to the number of scar segments and ≤4scar segments predicted recovery of global function. A lesser association is presented when viable myocardium was considered. In addition, Pegg et al [7] demonstrated that patients with ≥10 viable+normal segments could improve global LV function after CABG with a sensitivity and specificity of 95% and 75%, respectively. However, a small proportion of patients with viable myocardium often fail to improve global function post-CABG. Schinkel et al demonstrated that in general, multiple imaging techniques evaluating myocardial viability have a higher sensitivity (81–93%) but a lower specificity (58–80%) [25].Incorrect labeling of scar segments, incomplete revascularization, bypass graft failure, remodeling of LV and tethering by adjacent scar tissue may be the reasons of lower specificity [26].

The present study also demonstrated that a significant reverse remodeling and improvement of LV function were observed in patients with ≤4 scar segments. It is noteworthy that there was also statistical significance of improvement in global function or LV geometry at 6 months in patients with >4 scar segments. However, the mean difference of that improvement was less than 5%, thus fail to reach the standard of global functional recovery. Senior et al [18] demonstrated that even a little change in LV geometry was associated with improved outcome after CABG, and that any degree of LV reverse remodeling was likely to be associated with a survival benefit. From our experience, CABG might slightly improve both symptoms and cardiac function in patients with large amount of scar tissue. However, the effect of that inconspicuous improvement on the long-term prognosis of these patients is still unknown.

### Clinical aspects of survival analysis and prognostic value of scar tissue

Since CABG in patients with depressed LVEF as well as large scar tissue is associated with increased perioperative risk, it is therefore important to balance this risk with a potential benefit in survival by reduction of future CVEs after surgical revascularization. Earlier studies have evaluated the value of LGE to predict patients outcomes and reported that presence of larger scar tissue identifies patients with higher risk of CVEs [12], [13], [14], this appears to be in agreement with the results of the present study, where we observed that patients with ≤4 scar segments showed an excellent mid-term outcome (CVE-free survival of >96%), patients with >4 scar segments, however, was associated with a worse CVE-free survival, with a CVE rate of 19%, and two of them suffered from ventricular arrhythmias.

Several reasons might explain why scar tissue provided prognostic value beyond the parameters of LV function. Firstly, subclinical MI caused by ischemia may act as a certain substrate for malignant arrhythmia without impaired LV function [27]. Bello et al [14] further indicated that MI comprising greater than 10% of LV mass carries a two-fold risk of death and this risk may be due to the threshold-volume of necrosis tissue serving as a potentially fatal arrhythmogenic substrate. In addition to serving as a substrate for arrhythmias, the extent of myocardial scar is also a major determinant of LV remodeling which may finally lead to impaired LV function and heart failure [28]. Thirdly, the presence of myocardial necrosis may be a marker of coronary and peripheral artery atherosclerosis, which might be well recognized to be the major cause of CVEs [29].

### Study limitations

Several limitations of this study merit attention. The major limitation is that this is a single-center study with a relatively small number of patients. However, these numbers are comparable to prior similar studies, mainly because of CMR-method being quite time-consuming, financially-demanding and technically-challenging. Secondly, LGE-CMR was not performed before hospital discharge. Nevertheless, myocardial enzymes (creatine kinase, creatine kinase-MB, or troponin) and ECG were routinely checked during perioperative period. Thus, patients with procedure-related myocardial injury could be identified, which might influence the results of subsequent imaging analysis. Thirdly, we did not analyze the relationship between the bypassed vessel and segments with improved contractility simply because complete revascularization was performed in all patients and all the grafts were patent at 6 months evidenced by CTA scan.

## Conclusions

Based on our findings, we conclude that scar tissue on LGE-CMR is an independent negative predictor of cardiac functional recovery in patients with LV dysfunction undergoing surgical revascularization. Whether myocardial scar is a better predictor of functional recovery than viable myocardium remains to be tested in large-scale randomized clinical trials. This finding is of extremely important clinical significance, and may provide a simple approach to identify which patients with depressed LVEF are most likely to derive prognostic benefit from CABG.

## Supporting Information

Movie S1 **A patient with substantial scar tissue on LGE-CMR showed no evidence of functional improvement after coronary artery bypass grafting.**
(RAR)

Movie S2 **Global functional recovery was demonstrated in another patient with small scar area.**
(RAR)

## References

[1] Alderman EL, Fisher LD, Litwin P, Kaiser GC, Myers WO, et al (1983) Results of coronary artery surgery in patients with poor left ventricular function (CASS). Circulation 68: 785–795. 6352078 10.1161/01.cir.68.4.785

[2] Kim RJ, Wu E, Rafael A, Chen EL, Parker MA, et al (2000) The use of contrast-enhanced magnetic resonance imaging to identify reversible myocardial dysfunction. N Engl J Med 343: 1445–1453. 11078769 10.1056/NEJM200011163432003

[3] Gerber BL, Rousseau MF, Ahn SA, le Polain de Waroux JB, Pouleur AC, et al (2012) Prognostic value of myocardial viability by delayed-enhanced magnetic resonance in patients with coronary artery disease and low ejection fraction: impact of revascularization therapy. J Am Coll Cardiol 59: 825–835. 22361403 10.1016/j.jacc.2011.09.073

[4] Gerber BL, Darchis J, le Polain de Waroux JB, Legros G, Pouleur AC, et al (2010) Relationship between transmural extent of necrosis and quantitative recovery of regional strains after revascularization. JACC Cardiovasc Imaging 3: 720–730. 20633850 10.1016/j.jcmg.2010.03.008

[5] Sawada SG, Dasgupta S, Nguyen J, Lane KA, Gradus-Pizlo I, et al (2010) Effect of revascularization on long-term survival in patients with ischemic left ventricular dysfunction and a wide range of viability. Am J Cardiol 106: 187–192. 20599001 10.1016/j.amjcard.2010.03.014

[6] Bonow RO, Maurer G, Lee KL, Holly TA, Binkley PF, et al (2011) Myocardial viability and survival in ischemic left ventricular dysfunction. N Engl J Med 364: 1617–1625. 21463153 10.1056/NEJMoa1100358 PMC3290901

[7] Pegg TJ, Selvanayagam JB, Jennifer J, Francis JM, Karamitsos TD, et al (2010) Prediction of global left ventricular functional recovery in patients with heart failure undergoing surgical revascularisation, based on late gadolinium enhancement cardiovascular magnetic resonance. J Cardiovasc Magn Reson 12: 56. 20929540 10.1186/1532-429X-12-56 PMC2959056

[8] Wu YW, Huang PJ, Lee CM, Ho YL, Lin LC, et al (2005) Assessment of myocardial viability using F-18 fluorodeoxyglucose/Tc-99m sestamibi dual-isotope simultaneous acquisition SPECT: comparison with Tl-201 stress-reinjection SPECT. J Nucl Cardiol 12: 451–459. 16084434 10.1016/j.nuclcard.2005.04.007

[9] Kim HW, Farzaneh-Far A, Kim RJ (2009) Cardiovascular magnetic resonance in patients with myocardial infarction: current and emerging applications. J Am Coll Cardiol 55: 1–16. 20117357 10.1016/j.jacc.2009.06.059

[10] Bingham SE, Hachamovitch R (2011) Incremental prognostic significance of combined cardiac magnetic resonance imaging, adenosine stress perfusion, delayed enhancement, and left ventricular function over preimaging information for the prediction of adverse events. Circulation 123: 1509–1518. 21444886 10.1161/CIRCULATIONAHA.109.907659

[11] Hundley WG, Bluemke DA, Finn JP, Flamm SD, Fogel MA, et al (2010) ACCF/ACR/AHA/NASCI/SCMR 2010 expert consensus document on cardiovascular magnetic resonance: a report of the American College of Cardiology Foundation Task Force on Expert Consensus Documents. Circulation 121: 2462–2508. 20479157 10.1161/CIR.0b013e3181d44a8f PMC3034132

[12] Kwon DH, Halley CM, Carrigan TP, Zysek V, Popovic ZB, et al (2009) Extent of left ventricular scar predicts outcomes in ischemic cardiomyopathy patients with significantly reduced systolic function: a delayed hyperenhancement cardiac magnetic resonance study. JACC Cardiovasc Imaging 2: 34–44. 19356530 10.1016/j.jcmg.2008.09.010

[13] Kelle S, Roes SD, Klein C, Kokocinski T, de Roos A, et al (2009) Prognostic value of myocardial infarct size and contractile reserve using magnetic resonance imaging. J Am Coll Cardiol 54: 1770–1777. 19874990 10.1016/j.jacc.2009.07.027

[14] Bello D, Einhorn A, Kaushal R, Kenchaiah S, Raney A, et al (2011) Cardiac magnetic resonance imaging: infarct size is an independent predictor of mortality in patients with coronary artery disease. Magn Reson Imaging 29: 50–56. 20980115 10.1016/j.mri.2010.03.031

[15] Selvanayagam JB, Kardos A, Francis JM, Wiesmann F, Petersen SE, et al (2004) Value of delayed-enhancement cardiovascular magnetic resonance imaging in predicting myocardial viability after surgical revascularization. Circulation 110: 1535–1541. 15353496 10.1161/01.CIR.0000142045.22628.74

[16] Slart RH, Bax JJ, van Veldhuisen DJ, van der Wall EE, Dierckx RA, et al (2006) Prediction of functional recovery after revascularization in patients with coronary artery disease and left ventricular dysfunction by gated FDG-PET. J Nucl Cardiol 13: 210–219. 16580957 10.1007/BF02971245

[17] Cerqueira MD, Weissman NJ, Dilsizian V, Jacobs AK, Kaul S, et al (2002) Standardized myocardial segmentation and nomenclature for tomographic imaging of the heart: a statement for healthcare professionals from the Cardiac Imaging Committee of the Council on Clinical Cardiology of the American Heart Association. Circulation 105: 539–542. 11815441 10.1161/hc0402.102975

[18] Senior R, Lahiri A, Kaul S (2001) Effect of revascularization on left ventricular remodeling in patients with heart failure from severe chronic ischemic left ventricular dysfunction. Am J Cardiol 88: 624–629. 11564384 10.1016/s0002-9149(01)01803-3

[19] Schinkel AF, Poldermans D, Rizzello V, Vanoverschelde JL, Elhendy A, et al (2004) Why do patients with ischemic cardiomyopathy and a substantial amount of viable myocardium not always recover in function after revascularization? J Thorac Cardiovasc Surg 127: 385–390. 14762345 10.1016/j.jtcvs.2003.08.005

[20] Krittayaphong R, Laksanabunsong P, Maneesai A, Saiviroonporn P, Udompunturak S, et al (2008) Comparison of cardiovascular magnetic resonance of late gadolinium enhancement and diastolic wall thickness to predict recovery of left ventricular function after coronary artery bypass surgery. J Cardiovasc Magn Reson 10: 41. 18808697 10.1186/1532-429X-10-41 PMC2561019

[21] Bax JJ, Wijns W, Cornel JH, Visser FC, Boersma E, et al (1997) Accuracy of currently available techniques for prediction of functional recovery after revascularization in patients with left ventricular dysfunction due to chronic coronary artery disease: comparison of pooled data. J Am Coll Cardiol 30: 1451–1460. 9362401 10.1016/s0735-1097(97)00352-5

[22] Panza JA, Holly TA, Asch FM, She L, Pellikka PA, et al (2013) Inducible myocardial ischemia and outcomes in patients with coronary artery disease and left ventricular dysfunction. J Am Coll Cardiol 61: 1860–1870. 23500234 10.1016/j.jacc.2013.02.014 PMC3755503

[23] Camici PG, Prasad SK, Rimoldi OE (2008) Stunning, hibernation, and assessment of myocardial viability. Circulation 117: 103–114. 18172050 10.1161/CIRCULATIONAHA.107.702993

[24] Rizzello V, Poldermans D, Boersma E, Biagini E, Schinkel AF, et al (2004) Opposite patterns of left ventricular remodeling after coronary revascularization in patients with ischemic cardiomyopathy: role of myocardial viability. Circulation 110: 2383–2388. 15477413 10.1161/01.CIR.0000145115.29952.14

[25] Schinkel AF, Poldermans D, Elhendy A, Bax JJ (2007) Assessment of myocardial viability in patients with heart failure. J Nucl Med 48: 1135–1146. 17574986 10.2967/jnumed.106.038851

[26] Bax JJ, Schinkel AF, Boersma E, Elhendy A, Rizzello V, et al (2004) Extensive left ventricular remodeling does not allow viable myocardium to improve in left ventricular ejection fraction after revascularization and is associated with worse long-term prognosis. Circulation 110: II18–22. 15364832 10.1161/01.CIR.0000138195.33452.b0

[27] Cohn PF, Fox KM, Daly C (2003) Silent myocardial ischemia. Circulation 108: 1263–1277. 12963683 10.1161/01.CIR.0000088001.59265.EE

[28] Hombach V, Grebe O, Merkle N, Waldenmaier S, Hoher M, et al (2005) Sequelae of acute myocardial infarction regarding cardiac structure and function and their prognostic significance as assessed by magnetic resonance imaging. Eur Heart J 26: 549–557. 15713695 10.1093/eurheartj/ehi147

[29] McCrohon JA, Moon JC, Prasad SK, McKenna WJ, Lorenz CH, et al (2003) Differentiation of heart failure related to dilated cardiomyopathy and coronary artery disease using gadolinium-enhanced cardiovascular magnetic resonance. Circulation 108: 54–59. 12821550 10.1161/01.CIR.0000078641.19365.4C

